# A Rare Case of Dual Metachronous Primary Malignancies, Chronic Myeloid Leukemia, and Tongue Carcinoma in a Patient With Long-Standing Systemic Lupus Erythematosus: A Case Report and Review of Literature

**DOI:** 10.7759/cureus.56648

**Published:** 2024-03-21

**Authors:** Sitaraman BalajiSubramanian, Thuraya Al-Hajri, Namrata Satyapal, Mahdiya Al-Bulushi, Salma Mohammed Al Sheibani, Faisal Khamis Mubarak Al Kalbani, Maimuna Al-Saadi, Muhanna Nasser Al Musalhi, Humaid A Al Wahshi

**Affiliations:** 1 Department of Radiation Oncology, The Royal Hospital, Muscat, OMN; 2 Department of Hematology, The Royal Hospital, Muscat, OMN; 3 Department of Otolaryngology - Head and Neck Surgery, An Nahdha Hospital, Muscat, OMN; 4 Department of Pathology, Khoula Hospital, Muscat, OMN; 5 Department of Medicine, The Royal Hospital, Muscat, OMN

**Keywords:** autoimmune, radiotherapy, head and neck cancer, hematological malignancies, dasatinib, vmat, tongue carcinoma, chronic myeloid leukemia, systemic lupus erythematosus

## Abstract

Patients with long-standing autoimmune diseases like systemic lupus erythematosus (SLE) are at a higher risk of developing hematological malignancies. However, chronic myeloid leukemia (CML) has rarely been reported in patients with SLE. Advancements in medical diagnostics and treatment have led to the life expectancy of SLE and CML patients moving closer to that of the general population, and it is not uncommon to encounter more than one malignancy in a cancer survivor. Although squamous cell carcinoma (SCC) of the skin has been reported in CML patients, mucosal SCC of the head and neck has rarely only been reported in CML survivors. The objective of this case report is to share our experience in treating a patient with dual metachronous primary malignancies, CML, and tongue carcinoma, along with long-standing SLE, managed by a multidisciplinary team.

## Introduction

Systemic lupus erythematosus (SLE) is a multisystem autoimmune disorder occurring in women of reproductive age with a female-to-male prevalence ratio of 10:1 [1]. The hematological manifestations of SLE are anemia, leukopenia, thrombocytopenia, and clotting disorders. Additionally, SLE has been associated with hematological malignancies (HM), especially malignant lymphomas [2]. Although chronic myeloid leukemia (CML) is rarely associated with SLE, a Swedish study using population-based registers suggested that autoimmune diseases (AD) often precede CML diagnosis [3].

Available medical treatment has improved the prognosis of SLE, and the current five-year survival rate is more than 90% [4]. Similarly, following the introduction of tyrosine kinase inhibitor (TKI) imatinib and second-generation TKI-like nilotinib and dasatinib for CML, over the past two decades, five- and eight-year survival have reached 90% and 88%, respectively [5]. TKIs block the activity of specific tyrosine kinase enzymes, which are crucial for cell signaling pathways, particularly in cancer cells. Imatinib targets multiple tyrosine kinases, including the BCR-ABL protein, a key driver in CML. As a result, the life expectancy of CML patients is moving closer to that of the general population, which has significant clinical repercussions, especially in Asian countries, since the estimated age at diagnosis of CML in the Asian population is approximately a decade earlier than in the West [6]. In addition, studies have shown that TKI-treated CML patients have an increased risk of developing secondary malignancies with a predisposition to specific organs, including the head and neck, cervix, and prostate [7-9]. Moreover, a retrospective analysis using the Veterans Affairs (VA) Corporate Data Warehouse (CDW) suggested that prior HM was negatively associated with survival among patients with second primary head and neck squamous cell carcinoma (SCC) [10].

Surgery is the primary treatment modality for oral cavity cancers, followed by adjuvant treatment, either radiotherapy or chemoradiotherapy, depending on the risk assessed on final histopathology [11]. However, the decision to offer postoperative radiotherapy (PORT) to patients with connective tissue diseases remains challenging. Radiation oncologists frequently underuse radiotherapy due to fear of exacerbated acute and late side effects in treating patients with connective tissue diseases, especially scleroderma and active SLE [12]. Considerable advances and innovations in radiation technology like intensity-modulated radiotherapy, volumetric modulated arc therapy (VMAT), hybrid-VMAT, and image-guided radiotherapy can potentially reduce acute and late side effects and benefit patients with connective tissue diseases [13-15].

The present case report describes a young woman with long-standing SLE who developed CML. She was initially treated with dasatinib and went into remission. In remission, she developed a second primary tongue that required multidisciplinary treatment, including surgery and PORT.

## Case presentation

An 18-year-old female from Oman was evaluated for polyarthralgia, fever, malar rash, and fatigue in 2007 and diagnosed to have SLE based on the American College of Rheumatology criteria [16]. Laboratory investigations supported the diagnosis, and serological testing was positive for anti-DS-DNA, anti-Sm, anti-Ro, anti-RNP, and hypocomplementemia (low levels of C3 and C4). She also had autoimmune hemolytic anemia (direct Coombs positive) and leukopenia at diagnosis. Treatment was initiated with hydroxychloroquine (HCQ) and prednisolone. Subsequently, mycophenolate mofetil (MMF) was added to the regimen, and the steroid was tapered. Disease flares were controlled with intermittent increases in the dose of steroids.

In 2012, the treatment regimen was switched to azathioprine and HCQ during her conception (to minimize the risk of neonatal heart block considering anti-RO positivity), and she also received low-molecular-weight heparin (given a history of previous recurrent abortions). Following pregnancy, she was restarted on treatment with MMF, and HCQ was continued with the further addition of steroids in 2015.

**Figure 1:**
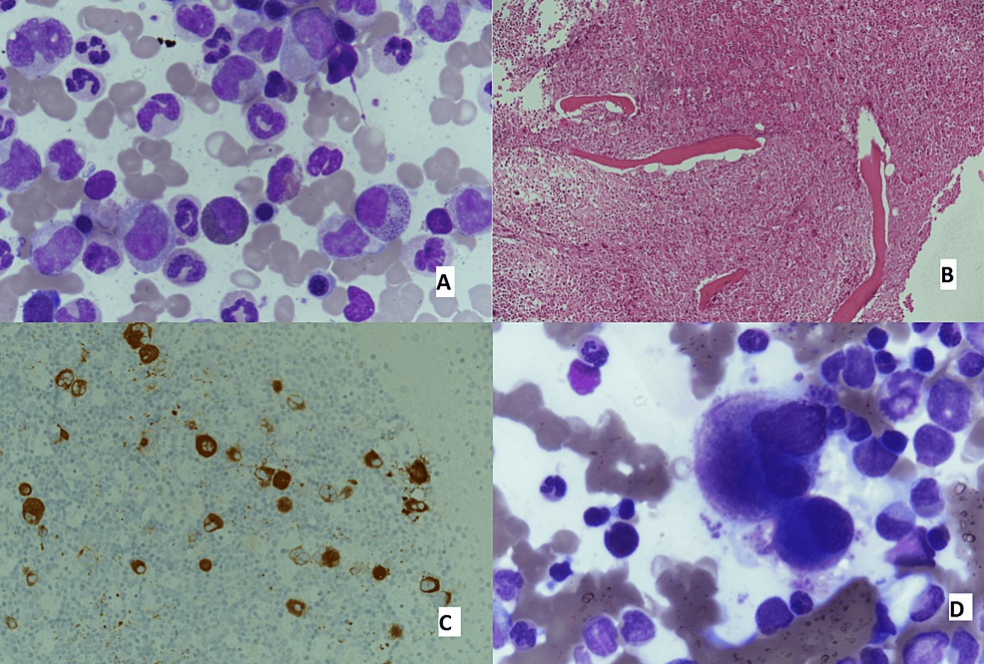
BM aspiration and trephine slides (A) BM aspiration showing myeloid hyperplasia in CML with the peak of myelocytes. (B) BM trephine showing granulocytic hyperplasia. (C) BM trephine: immunohistochemical stains; x400-CD61 shows hyperplasia of megakaryocytes. (D) High-power view of BM trephine at 400x magnification showing megakaryocytic hyperplasia with dwarf platelets. BM: bone marrow

During FUP, routine lab tests in November 2016 revealed leucocytosis, and further evaluation confirmed CML. Additionally, the bone marrow (BM) examination showed hypercellularity; IHC was positive for CD61 and MPO, and molecular analysis (FISH panel utilizing LSI ABL1/BCR t(9;22) dual color translocation probe) revealed a typical BCR-ABL t(9;22) (q34;q11) reciprocal translocation resulting in the Philadelphia chromosome. Figure 1 shows the BM aspiration and trephine slides.

The pre-treatment SOKAL score was 0.85 (intermediate risk). The patient was started on dasatinib 100 mg. While on treatment for CML, she had an exacerbation of synovitis and was prescribed rituximab to control symptoms. Although remission attained initially was transient due to poor treatment compliance, with subsequent counseling, she sustained hematologic, cytogenetic, and molecular remission at the end of twelve months.

**Figure 2:**
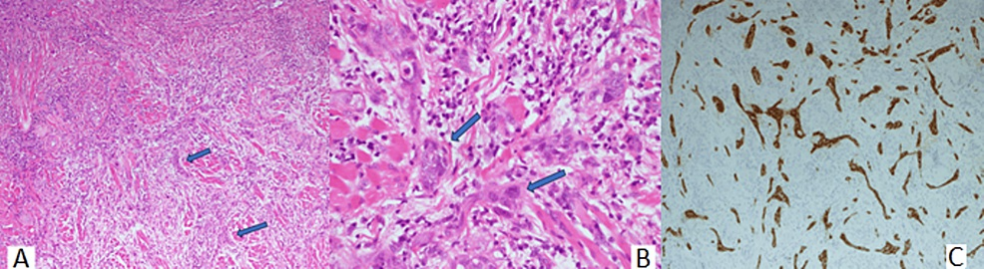
Tongue biopsy result (A) Low power (4x, H&E) of a poorly differentiated tumor composed of nests and cords of tumor cells (arrows). (B) High power (40x, H&E) showing tumor nests and cords composed of atypical cells with irregularly enlarged hyperchromatic nuclei (arrows). (C) AE1/AE3 immunostain highlighting the epithelial nests and cords

While her CML was in remission five years into treatment with dasatinib, along with MMF, HCQ, and steroids for SLE, in October 2021, she developed multiple oral mucosal ulcerations, for which an ENT opinion was sought. There was a non-healing ulcerative lesion along the left lateral border of the tongue, which was biopsied in December 2021. Histopathology was suggestive of a moderate-to-poorly differentiated SCC. Figure 2 shows the biopsy results.

CT imaging revealed a lesion in the left lateral border of the tongue with no significant neck nodes. She was subjected to wide local excision of the left lateral border tongue lesion and left modified radical neck dissection in early January 2022, and the final pathological staging was pT3N0 with clear margins. In addition, the maximum tumor size was 3.6 cm, the DOI was 9 mm, and the worst pattern of invasion was 5, focal perineural invasion with no lymphovascular space invasion. After the MDT discussion, the patient was offered PORT due to high-risk features. In addition, the possibility of increased radiotherapy-induced reactions was discussed with the patient's family. The patient had a pre-radiotherapy dental evaluation and maintained good oral hygiene. There was minimal plaque accumulation and no signs of gingivitis. The teeth were present and intact, with no fractured cusps or sharp edges.

The patient was immobilized supine with a thermoplastic mask before undergoing a planned CT scan, and VMAT-based plans were optimized in the Eclipse treatment planning system using 6 MV photons for multiple arcs for a Truebeam STx (Varian Medical Systems, USA) equipped with a high-definition multileaf collimator with 120 leaves. A dose of 60 Gy in 30 fractions was given to the primary tumor bed, while the bilateral neck (level of nodes included I-IV) was given a dose of 54 Gy in 30 fractions using a simultaneous integrated boost technique with VMAT. Table 1 shows the dose parameters for organs at risk for the VMAT plans [17].

**Table 1:** Dose parameters to organs at risk for the VMAT plans OAR: organs at risk, QUANTEC: Quantitative Analyses of Normal Tissue Effects in the Clinic, PTV: planning target volume, Gy: Gray

OAR	Dose parameter	Recommended values (QUANTEC)	Achieved values
PTV coverage	D95%	-	95%
Spinal cord	Dmax	≤50 Gy	40.5 Gy
Parotid right	Dmean	≤26 Gy	19.9 Gy
Parotid left	Dmean	≤26 Gy	26.8 Gy
Mandible	Dmax	≤70 Gy	63.2 Gy
Pharyngeal constrictors	Dmean	≤50 Gy	30.9 Gy
Larynx	Dmean	≤44 Gy	34.1 Gy
Esophagus	Dmean	≤34 Gy	33.3 Gy

**Figure 3:**
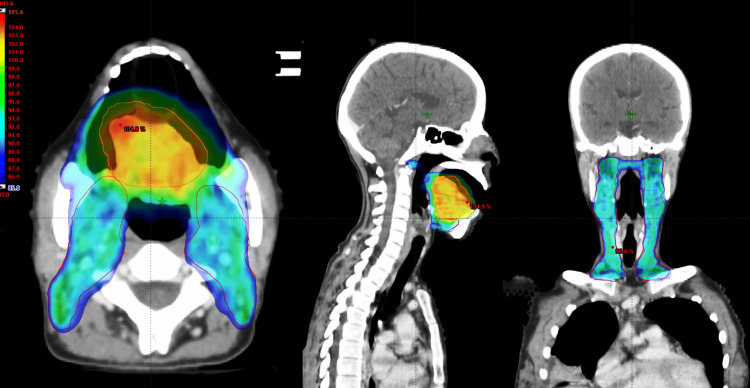
VMAT plan dose distribution to planning target Dose in the color wash showing 85% isodose

Figure 3 shows the VMAT plan dose distribution to the planned target.

The patient developed grade 2 mucosal and skin reactions during radiotherapy, which were managed conservatively. According to institutional protocol, the patient was advised to come for routine clinical-radiological follow-up visits every three months for the first two years after radiotherapy and every six months for the next three years. Clinical follow-up every three months post-radiotherapy revealed no unusual local skin or mucosal toxicity. She is currently on regular follow-up, and there has been no clinical-radiological evidence of locoregional disease in the tongue for two years. Still, she experiences fluctuating symptoms of SLE, affecting her quality of life.

## Discussion

SLE is a chronic and complex multisystem autoimmune disorder characterized by intrinsic immunological dysregulation and lymphocyte hyperactivity. Although the underlying molecular mechanisms involved in pathogenesis are largely unknown and are an active area for research, they are generally considered due to intrinsic dysregulation and exposure to immunomodulatory medications and viruses. Table 2 includes population-based and case-control research studies examining the relationship between autoimmune disorders and HM [2,3,18,19].

**Table 2:** Studies examining the relationship between autoimmune disorders and HM SLE: systemic lupus erythematosus, NHL: non-Hodgkins lymphoma, CML: chronic myeloid leukemia, HM: hematological malignancies, AD: autoimmune diseases

Authors	Publication year	Study type	Conclusion
Bernatsky et al. [2]	2009	Case-control	Cancer risk in SLE was slightly increased, especially NHL
Löfström et al. [18]	2013	Cohort	Identified leukopenia as a risk factor for myeloid leukemia in SLE patients
Paul et al. [20]	2014	Case report	Reported a single case of CML associated with SLE
Gunnarsson et al. [3]	2016	Case-control	AD often precedes CML diagnosis
Zhang et al. [19]	2022	Case-control	Older age at SLE diagnosis as a risk factor for HM

Overall, these studies suggest an increased risk of HM in SLE patients, especially non-Hodgkins lymphoma. In addition, a retrospective study suggested older age at SLE diagnosis as a risk factor for HM [19]. However, our patient was diagnosed early, when she was eighteen. The factors contributing to the development of HM in SLE are unclear; genetic predisposition, cumulative disease activity, medications, or damage to BM due to SLE are sometimes implicated.

In long-term survivors, studies have shown persistent leucopenia to be a high-risk factor for developing myeloid leukemia, which was seen in our patient [16,20]. In a large case-control study in a population with primary AD, azathioprine exposure was associated with a sevenfold risk for myeloid neoplasm. In addition, the median time from AD onset to myeloid neoplasm diagnosis was eight years [21]. Our patient was on azathioprine during her disease course for four years, and the time from the onset of SLE symptoms to the diagnosis of CML was nine years.

The evolution of the pathophysiology of CML indicated that the chromosomal defect of the Philadelphia chromosome resulted from a reciprocal translocation in a hematopoietic cell between the long arms of the 9th and 22nd chromosomes. Subsequently, the BCR-ABL1 fusion gene was characterized. This fusion gene is encoded into a protein called P210BCR-ABL1, a tyrosine kinase enzyme that phosphorylates from ATP to a tyrosine. The introduction of first-generation TKI-Imatinib and, subsequently, second-generation TKI have drastically improved survival, and currently, the five-year overall survival is 90% [5].

The European LeukemiaNet recommends imatinib, dasatinib, or nilotinib as a first-line treatment [22]. Achieving an early, complete cytogenetic response and a major molecular response within 12 months is an important treatment goal since it is associated with a low risk of long-term progression [23]. Therefore, dasatinib was initiated for our patient, and she achieved a complete cytogenetic and major molecular response.

In the pre-Imatinib era, a population-based study analyzing data from the Swedish cancer registry assessed the incidence rate of second primary cancers (all sites and specific sites) in CML patients and reported an 80% relative increase in cancer incidence compared to the general population [24]. Long-term survival in CML patients in the TKI era has led to a focus on research to prove the causal association between TKI and the development of the second primary. However, retrospective and cohort studies have not shown evidence for the same [9,25,26].

Table 3 includes observational and case studies reporting the risk of second malignancies in general and that of head and neck cancers in CML patients.

**Table 3:** Literature review reporting risk/incidence of second malignancies in general and that of head and neck cancers in CML patients CML: chronic myeloid leukemia, TKI: tyrosine kinase inhibitors, HM: hematological malignancies

Authors	Publication year	Study type	Conclusion
Rebora et al. [24] (pre-imatinib pts)	2010	Population-based	Suggested a higher incidence of second malignancies in CML patients
Verma et al. [25] (imatinib treated)	2011	Retrospective single center	No evidence that exposure to TKIs increases the risk of developing second cancers
Budrukkar et al. [8]	2011	Retrospective case series	Suggested that mucosal cancers in the head and neck can occur in long-term survivors of CML. In addition, tend to be aggressive
Miranda et al. [26]	2016	Observational study	Found no increased risk for secondary malignancies in CML patients
Gunnarsson et al. [9]	2016	Cohort study (population-based)	Found that rather than TKI, it was CML itself linked to increased risk of gastro-intestinal and nose and throat cancer
Mowery et al. [10]	2019	Retrospective	Found a positive association between HM (not specific to CML) and subsequent head and neck cancer

The cause of the development of head and neck SCCs in this subset of patients remains unknown. However, in one case series of seven patients with SCC of the head and neck region with prior CML from India, it was suggested that mucosal cancers could develop in long-term CML survivors, and they tend to be more aggressive, although five of the seven patients had a history of tobacco use [8]. Additionally, a retrospective analysis using the VA CDW showed that prior HM was associated with a 1.6-fold increased risk of aerodigestive tract cancer and was negatively associated with survival among patients with second primary head and neck SCC [10]. Moreover, the mean time from HM diagnosis to head and neck cancer diagnosis was four years; our patient's interval was five years.

The adjuvant treatment for oral cavity cancers consists of radiotherapy or chemoradiotherapy, as guided by the adverse risk features in the final histopathology report [11]. However, collagen vascular disorder has long been considered a relative contraindication to radiotherapy, despite retrospective and case-control studies failing to provide evidence of a prohibitive increase in high-grade toxicity after radiotherapy for patients with SLE [27,28].

Recent research from Ireland raised the issue of therapeutic nihilism among physicians regarding the prognosis of cancer patients [29]. This attitude holds even for patients who require radiotherapy for connective tissue diseases. Radiation oncologists and other physicians alike are reluctant to offer radiation treatment due to fear of exacerbating acute and late side effects. Therefore, in addition to new-generation radiotherapy techniques and well-designed prospective studies, educating oncologists and other physicians is required to ensure that patients are not denied radiotherapy if necessary.

In spite of the complexity of our patient's care, involving different specialists in rheumatology, hematology, oncology, and head and neck surgery as part of a multidisciplinary care team, our patient was treated based on evidence-based guidelines, and the outcome was positive.

## Conclusions

Asians are diagnosed with CML a decade earlier than the Western population, and these long-term survivors must have a comprehensive multisystem cancer screening and a follow-up plan to identify signs of second primary tumors because early diagnosis and treatment can significantly affect clinical outcomes. This case report highlights that an evidence-based, multidisciplinary care approach can improve outcomes for patients with multiple metachronous primary malignancies with ADs like SLE.
